# Self-assembled FGF21 nanoparticles alleviate drug-induced acute liver injury

**DOI:** 10.3389/fphar.2022.1084799

**Published:** 2023-01-10

**Authors:** Zhiwei Huang, Hengcai Wang, Changju Chun, Xinze Li, Shihao Xu, Yingzheng Zhao

**Affiliations:** ^1^ Department of pharmaceutics, School of Pharmaceutical Sciences, Wenzhou Medical University, Wenzhou, China; ^2^ College of Pharmacy, Research Institute of Pharmaceutical Sciences, Chonnam National University, Gwangju, South Korea; ^3^ Department of Emergency, The First Affiliated Hospital of Wenzhou Medical University, Wenzhou, China; ^4^ Department of Ultrasonography, The First Affiliated Hospital of Wenzhou Medical University, Wenzhou, China

**Keywords:** fibroblast growth factor 21, nanoparticles, liver-targeted therapy, oxidative stress, inflammation

## Abstract

Acetaminophen (N-acetyl-p-aminophenol, APAP) is a common antipyretic agent and analgesic. An overdose of APAP can result in acute liver injury (ALI). Oxidative stress and inflammation are central to liver injury. N-acetylcysteine (NAC), a precursor of glutathione, is used commonly in clinical settings. However, the window of NAC treatment is limited, and more efficacious alternatives must be found. Endogenous cytokines such as fibroblast growth factor (FGF) 21 can improve mitochondrial function while decreasing intracellular oxidative stress and inflammatory responses, thereby exhibiting antioxidant-like effects. In this study, self-assembled nanoparticles comprising chitosan and heparin (CH) were developed to deliver FGF21 (CH-FGF21) to achieve the sustained release of FGF21 and optimize the *in vivo* distribution of FGF21. CH-FGF21 attenuated the oxidative damage and intracellular inflammation caused by APAP to hepatocytes effectively. In a murine model of APAP-induced hepatotoxicity, CH-FGF21 could alleviate ALI progression and promote the recovery of liver function. These findings demonstrated that a simple assembly of CH nanoparticles carrying FGF21 could be applied for the treatment of liver diseases.

## 1 Introduction

Drug-induced liver injury is one of the most serious adverse drug reactions, and can lead to liver dysfunction and lethal complications. In general, acetaminophen (APAP) is used as an antipyretic and painkiller. An overdose of APAP is a prevalent cause of acute liver injury (ALI) and even acute liver failure (ALF) in humans (Chao et al., 2018). Patients with more severe liver diseases (e.g., ALF) have significantly higher costs for treatment (e.g., liver transplantation). Early intervention against ALI prevents the consequences of more advanced liver disorders and reduces the associated economic burden.

APAP-induced oxidative stress and the associated inflammatory response in the liver are commonly implicated in the etiology of ALI, and cause extensive necrosis of liver tissue (Yan et al., 2018; Ramachandran and Jaeschke, 2019; Jaeschke et al., 2021). A toxic dose of APAP produces an excessive amount of N-acetyl-p-benzo-quinone imine (NAPQI). This action results in severe depletion of glutathione (GSH), lipid peroxidation, reactive oxygen species (ROS) release, and inflammatory reactions in the liver that, ultimately, lead to damage and necrosis of hepatocytes (Ali et al., 2021; Wang et al., 2021). Therefore, prompt and efficacious management of oxidative stress and inflammation in the injured liver are crucial tools in ALI treatment.

N-acetylcysteine (NAC) encourages the recovery of the hepatic level of GSH, and is a therapeutically recognized antidote. However, NAC has negative effects (e.g., anaphylactic reactions and fluid overload) and the standard therapeutic dose of NAC may not provide the desired effects for patients with ALI (Pakravan et al., 2008). As a result, the quest for and development of safe and efficacious therapeutic agents against drug-induced ALI has become a research priority.

Fibroblast growth factor (FGF) 21 is a peptide hormone comprising 181 amino acids. It is generated mainly by hepatocytes and is crucial for maintaining the metabolic balance of energy, physiological activities, and insulin sensitivity in the body (Fisher and Maratos-Flier, 2016). Exogenous administration of FGF21 has been shown to be efficacious in the treatment of metabolic diseases such as type-2 diabetes mellitus, fatty liver, and cardiovascular disease. FGF21 reduces the accumulation of circulating levels of hepatic triglycerides and maintains the homeostasis of the metabolism of glucose and lipids in the body. FGF21 has antioxidant and anti-inflammatory properties, and is used to treat various diseases (Geng et al., 2020). Recent studies have shown that FGF21 can protect against mitochondrial damage in hepatocytes caused by iron overload by stimulating the ubiquitination and degradation of heme oxygenase-1 and activation of nuclear factor E2-related factor 2 (Nrf2) to suppress ferroptosis in hepatocytes (Wu et al., 2021). In the treatment of non-alcoholic steatohepatitis, FGF21 and its analogs have shown encouraging outcomes, and FGF21 can prevent the deterioration of steatosis, inflammation, hepatocyte injury, fibrosis, and cirrhosis in observed non-alcoholic steatohepatitis (Tucker et al., 2019). In addition, FGF21 therapy can ameliorate carbon tetrachloride-induced ALI by activating the sirtuin type 1-mediated autophagy pathway (Yang et al., 2022). Those findings indicate that FGF21 has considerable value in the treatment of liver diseases. Unfortunately, FGF21 has poor bioavailability because of its weak absorption, relatively short half-life, and instability (Gao et al., 2021), which restricts its therapeutic potential against ALI. Despite the powerful physiological regulatory capacities of FGF21, few drug carriers have been designed to address these drawbacks of FGF21.

Nanotechnology-based drug-delivery systems (DDSs) have demonstrated notable advantages over free therapeutic agents in the treatment of liver disorders (Reddy and Couvreur, 2011). Due to the physiological structure of the liver, drugs in nanoparticle form are more likely to accumulate in this organ (Liu et al., 2018; Jin et al., 2020). Thanks to a meshwork of reticuloendothelial cells, a network of liver sinusoids can capture many nanoparticles in the blood circulation (Jenne and Kubes, 2013). In addition, the presence of a mononuclear phagocyte system (macrophages) in the liver can take-up nanoparticles (Wilhelm et al., 2016). Taking advantage of these features, several nanomedicines have been developed and applied to the treatment and diagnosis of liver diseases (Wang et al., 2015; Poilil Surendran et al., 2017; Chi et al., 2020). The enhanced permeability and retention (EPR) effect of the inflamed liver also increases nanoparticle retention in liver tissue (Kiessling et al., 2014). Studies have shown that ruthenium nanoparticles with antioxidant activity can be exploited to treat APAP-induced ALI (Xia et al., 2022). In addition, nanoparticles formed by coupling cyclic phenylboronic acid with baicalein can target the liver for efficacious treatment of hepatitis (Zhang et al., 2021). Although those novel nanomedicines show excellent liver-targeting properties and good therapeutic effects, their safety and toxic effects merit further evaluation (Zhao and Castranova, 2011). In contrast, the selection of biocompatible substances for the development of nano-DDSs is more likely to be applied.

By harnessing the passive targeting capabilities of nanoparticles to the liver, we prepared self-assembled chitosan/heparin nanoparticle-loaded FGF21 (CH-FGF21) that could target the liver after systemic administration. CH-FGF21 was prepared readily and was cost-effective with high biocompatibility. CH-FGF21 enhanced the efficiency of FGF21 internalization by hepatocytes, transported FGF21 to the APAP-injured liver preferentially, and increased the FGF21 concentration in the inflamed liver, thereby showing promising therapeutic effects in an ALI model. Our study offers a potential strategy for enhancing the efficacy of FGF21 for the treatment of liver diseases.

## 2 Materials and methods

### 2.1 Materials, cells, and animals

The protocol for experiments and procedures involving animals was approved by the Animal Care and Use Committee of Wenzhou Medical University (Wenzhou, China). Experiments were carried out in accordance with the National Research Council’s Guide for the Care and Use of Laboratory Animals.

Cell counting kit-8 (CCK-8) was obtained from Yeasen Biotechnology (Shanghai, China). Kits for superoxide dismutase (SOD) activity, malondialdehyde (MDA) assay, GSH assay, glutathione peroxidase (GSH-PX) assay, hematoxylin and eosin (H&E) staining, as well as 4′,6-diamidino-2-phenylindole (DAPI) solution were purchased for Solarbio Science & Technology (Beijing, China). A ROS assay kit as well as antibodies against myeloperoxidase (MPO, AF7494) and tumor necrosis factor (TNF)-α; AF8208) were obtained from Beyotime Biotechnology (Shanghai, China). Cell culture plates, coverslips, and centrifuge tubes were sourced from NEST Biotechnology (Wuxi, China). Enzyme-linked immunosorbent assay (ELISA) kits for interleukin (IL)-6 (EK206), IL-1β (EK201B), MPO (EK2133), and TNF-α (EK282) were obtained from Multisciences Biotechnology (Hangzhou, China). Antibody against Ki67 (ab16667) was obtained from Abcam (Cambridge, United Kingdom).

A normal hepatocyte cell line from mice (AML12) was purchased from Procell Life Science & Technology (Wuhan, China). Cells were cultured in Dulbecco’s modified Eagle’s medium/Nutrient Mixture F-12 (DMEM/F-12) supplemented with 10% (*v/v*) fetal bovine serum and 1% (*v/v*) penicillin–streptomycin in an incubator with an atmosphere of 5% CO_2_ at 37°C.

Male C57BL/6 mice (22–26 g) were purchased from Shanghai Laboratory Animal Center (Shanghai, China).

### 2.2 Preparation of CH-FGF21

Chitosan/heparin (CH) nanoparticles were prepared using a physical self-assembly method (Liu et al., 2007). Briefly, a chitosan solution (2 mg/mL, in an aqueous solution of 1% acetic acid) and heparin (1 mg/mL, in deionized water) was prepared, mixed, and stirred for 30 min. Then, CH nanoparticles were obtained by centrifugation (15,000 rpm, 5 min, room temperature). To obtain CH-FGF21, FGF21 (10–200 μg/mL) was added to CH nanoparticles and stirred in deionized water, and the unloaded FGF21 was removed by centrifugation.

### 2.3 Characterization of CH-FGF21

The distribution of particle sizes, polydispersity index (PDI), and zeta potential of CH nanoparticles and CH-FGF21 were detected by a particle size analyzer (Anton-Paar, Graz, Austria). Changes in the size of CH nanoparticles and CH-FGF21 over 5 days were also recorded. The release behavior of FGF21 and CH-FGF21 was tested utilizing the dialysis method in phosphate-buffered saline (PBS). Briefly, FGF21 and CH-FGF21 were transferred to a dialysis bag (100 kDa) and swirled constantly. Then, the samples in the dialysis bag were removed to measure the FGF21 concentration and replenished with an equal volume of fresh PBS at predefined time intervals. The ultraviolet–visible spectra of CH nanoparticles, FGF21, and CH-FGF21 were recorded by a spectrophotometer (Varian, Palo Alto, CA, United States). After centrifugation, the FGF21 concentration in various CH-FGF21 formulations was measured to calculate the encapsulation efficiency (EE%) of CH-FGF21 using the following equation:

EE% = [(total mass of FGF21—mass of FGF21 in supernatant)/total mass of FGF21] × 100%

The drug-loading capacity (LC%) of CH-FGF21 was calculated using the following equation:

LC% = [(total mass of FGF21—mass of FGF21 in supernatant)/total mass of CH-FGF21] × 100%

Morphological examination of CH nanoparticles and CH-FGF21 was undertaken by transmission electron microscopy (TEM, Tokyo, Japan).

### 2.4 Cellular uptake

To compare the cellular uptake of FGF21 and CH-FGF21, Cy5 (fluorescent compound used commonly to label proteins) was conjugated to FGF21 and CH-FGF21, as reported previously (Li et al., 2022). Briefly, an FGF21 solution (1 mg/mL) and Cy5 solution (10 μg/mL) were mixed and stirred for 2 h. Then, unconjugated Cy5 was removed by dialysis and the obtained mixture was lyophilized. Cy5-labeled FGF21 was used to prepare fluorescent CH-FGF21. AML12 cells were seeded in a confocal dish. After 12 h, AML12 cells were treated for 2 h or 4 h with Cy5-labeled FGF21 (100 ng/mL) and CH-FGF21 (100 ng/mL), respectively. Subsequently, AML12 cells were washed thrice with PBS and stained with DAPI. Fluorescent images of AML12 cells were captured by confocal laser scanning microscopy using a setup from Nikon (Tokyo, Japan).

### 2.5 *In vitro* cytotoxicity assay

The CCK-8 assay was conducted to assess the cytotoxicity of FGF21 and CH-FGF21. AML12 cells were cultured in 96-well plates (2.0 × 10^3^ cells/well) for 24 h at 37°C. AML12 cells were treated with fresh DMEM/F-12 containing FGF21 (2.5–100 ng/mL) or CH-FGF21 (2.5–100 ng/mL). After incubation for 24 h, CCK-8 reagent (10 µL) was added to each well and the absorbance was measured at 450 nm with a microplate reader to calculate the cell viability.

### 2.6 Cytoprotective effects of CH-FGF21

To evaluate the cytoprotective property of CH-FGF21, AML12 cells were exposed to APAP (7.5 mmol/L) for 4 h and then incubated with FGF21 (100 ng/mL) or CH-FGF21 (100 ng/mL), respectively, for 24 h. After incubation, the intracellular ROS level of each group was labeled by a ROS probe (dichloro-dihydro-fluorescein diacetate) and observed under a fluorescence microscope (Olympus, Tokyo, Japan). Levels of SOD, GSH-PX, and MDA in the cells of each group were measured using the appropriate detection kits. Secretion of TNF-α and IL-6 in cells was detected by ELISA kits. The GSH level in AML12 cells of each group after APAP stimulation at different time points was measured to assess the effects of CH-FGF21 on enhancing cellular resistance to APAP damage. To evaluate the anti-inflammatory effects of CH-FGF21 upon macrophages, murine macrophage cells (RAW 264.7) were treated according to the same procedure, and levels of IL-6 and TNF-α in each group measured.

### 2.7 Biodistribution of CH-FGF21

Mice suffering from ALI were given intravenous injections of Cy5-labeled FGF21 or Cy5-labeled CH-FGF21 for a biodistribution study. The major organs of the mice mentioned above were collected 2 h and 4 h post-injection. The fluorescence intensity of these organs was analyzed by an imaging system (CLS136340; PerkinElmer, Waltham, MA, United States).

### 2.8 APAP-induced ALI model and assessment of CH-FGF21 efficacy

At the end of food restriction, mice received injections of APAP (500 mg/kg, i.p.) to create the ALI model. After 4 h, ALI mice received different treatments (Xia et al., 2022). These mice were assigned randomly to three groups of eight: ALI + saline; ALI + FGF21 (1 mg/kg, i.v.); ALI + CH-FGF21 (1 mg/kg, i.v.). Mice treated with physiologic saline were used as a control group. In addition, ALI mice were treated with NAC (100 mg/kg, i.v.) and CH nanoparticles (1 mg/kg, i.v.) as controls. Mice were killed 24 h after different treatments. The blood and liver tissue of mice were collected for analysis. Levels of aspartate aminotransferase (AST) and alanine aminotransferase (ALT) in the blood of mice of each group were measured 0, 1, 3, 6, 12, and 24 h after treatment by an automatic biochemical analyzer (AU480; Beckman Coulter, Fullerton, CA, United States) to assess the liver function of mice. In addition, the contents of pro-inflammatory cytokines (TNF-α, IL-1β, IL-6), MPO, MDA, SOD, and GSH in liver tissues from each group of mice were measured by the respective reagent kits.

### 2.9 Histology

Harvested liver tissues were fixed with 4% paraformaldehyde for 24 h, dehydrated, embedded in paraffin, and sectioned (thickness = 5 µm) for H&E staining. For the immunostaining assay, sections of liver tissue were stained with antibodies against TNF-α, MPO, and Ki67. Then, N,N-dimethyl-4-aminoazobenze was utilized to visualize positive areas. Stained tissue sections were observed under an optical microscope (Olympus) and the images recorded.

### 2.10 Statistical analyses

Data are the mean ± standard deviation. Statistical analysis was undertaken using the Student’s *t*-test and one-way analysis of variance. *p* ≤ .05 was considered significant.

## 3 Results

### 3.1 Characterization of CH-FGF21

**FIGURE 1:**
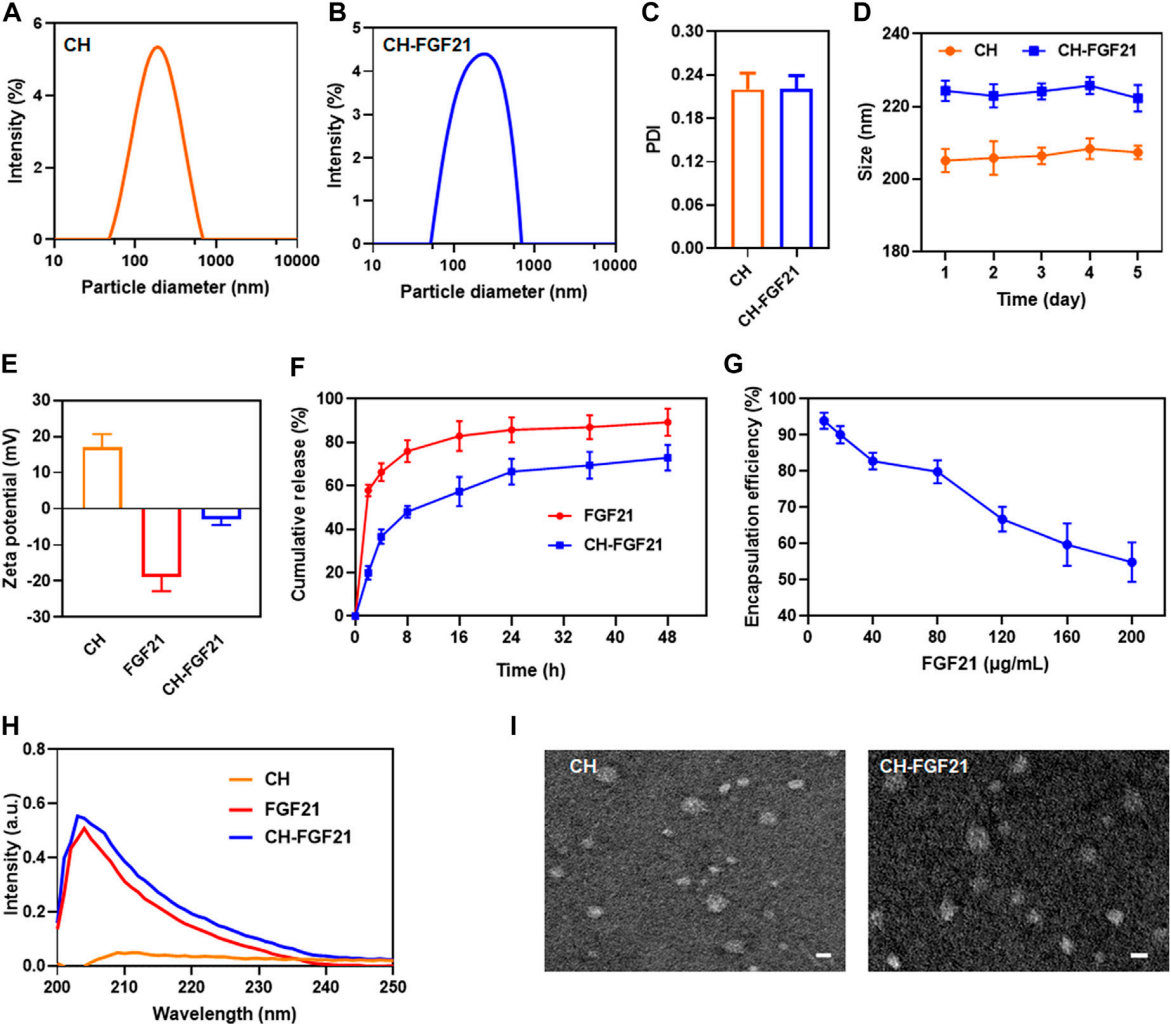
Characterization of CH-FGF21. The size distribution of **(A)** CH nanoparticles and **(B)** CH-FGF21. **(C)** Polydispersity index (PDI) of CH nanoparticles and CH-FGF21. **(D)** Size change of CH nanoparticles and CH-FGF21 over 5 days. **(E)** Zeta potential of CH nanoparticles, FGF21, and CH-FGF21. **(F)** *In vitro* release of FGF21 and CH-FGF21. **(G)** Encapsulation efficiency of CH-FGF21. **(H)** Ultraviolet-visible (UV-vis) spectra of CH nanoparticles, FGF21, and CH-FGF21. **(I)** Transmission electron micrographs of CH nanoparticles and CH-FGF21. Scale bar = 200 μm. Data are the mean ± SD (*n* = 3). *p ≤ .05, **p ≤ .01.

The distributions of size of CH nanoparticles and CH-FGF21 are shown in Figures 1A, B. The mean hydrodynamic size of CH nanoparticles and CH-FGF21 was 205.1 ± 3.2 nm and 224.3 ± 2.8 nm, respectively. The diameter of CH-FGF21 was marginally larger than that of CH nanoparticles, which mainly contributed to FGF21 attachment. The PDI of CH nanoparticles and CH-FGF21 was .220 ± .023 and .221 ± .018, respectively, which suggested that both had good dispersibility (Figure 1C). The size of CH nanoparticles and CH-FGF21 did not vary considerably over the continuous 5-day monitoring period, thereby indicating that CH nanoparticles and CH-FGF21 were stable (Figure 1D). The positively charged CH nanoparticles and negatively charged FGF21 formed slightly negatively charged CH-FGF21 under the influence of electrostatic action (Figure 1E). *In vitro* FGF21 release from CH-FGF21 was measured and compared with that of free FGF21. FGF21 showed rapid release during the first 4 h (>60%), whereas CH-FGF21 required 24 h to reach the same level of release, indicating that CH-FGF21 was a sustained-release system (Figure 1F). The *in vitro* release of CH-FGF21 over 120 h was also assessed. CH-FGF21 release reached >95% after 120 h (i.e., the vast majority of CH-FGF21 was released after 120 h) (Supplementary Figure S1). To obtain CH-FGF21 by mixing CH nanoparticles and different concentrations of FGF21 (10–200 μg/mL), samples were centrifuged, the supernatant was gathered, and the amount of FGF21 was measured. EE% decreased gradually as the FGF21 concentration increased. Notably, at FGF21 concentrations of 10–40 μg/ml and 80–200 μg/mL, EE% decreased significantly with increasing FGF21 concentration whereas, at a FGF21 concentration of 40–80 μg/mL, the declining trend of EE% was more moderate than that of other concentration intervals (Figure 1G). Based on this result, 80 μg/ml of FGF21 was used to prepare CH-FGF21, and the EE% and LC% of CH-FGF21 was 79.9 ± 3.2% and 3.1 ± .6%, respectively. The ultraviolet–visible spectrum of FGF21 and CH-FGF21 displayed a distinctive peak at 203 nm (Figure 1H). TEM images of CH nanoparticles and CH-FGF21 revealed a spherical structure (Figure 1I). Taken together, these findings verified the successful preparation of self-assembled CH-FGF21 nanoparticles.

### 3.2 Cellular uptake and cytotoxicity of CH-FGF21

**FIGURE 2:**
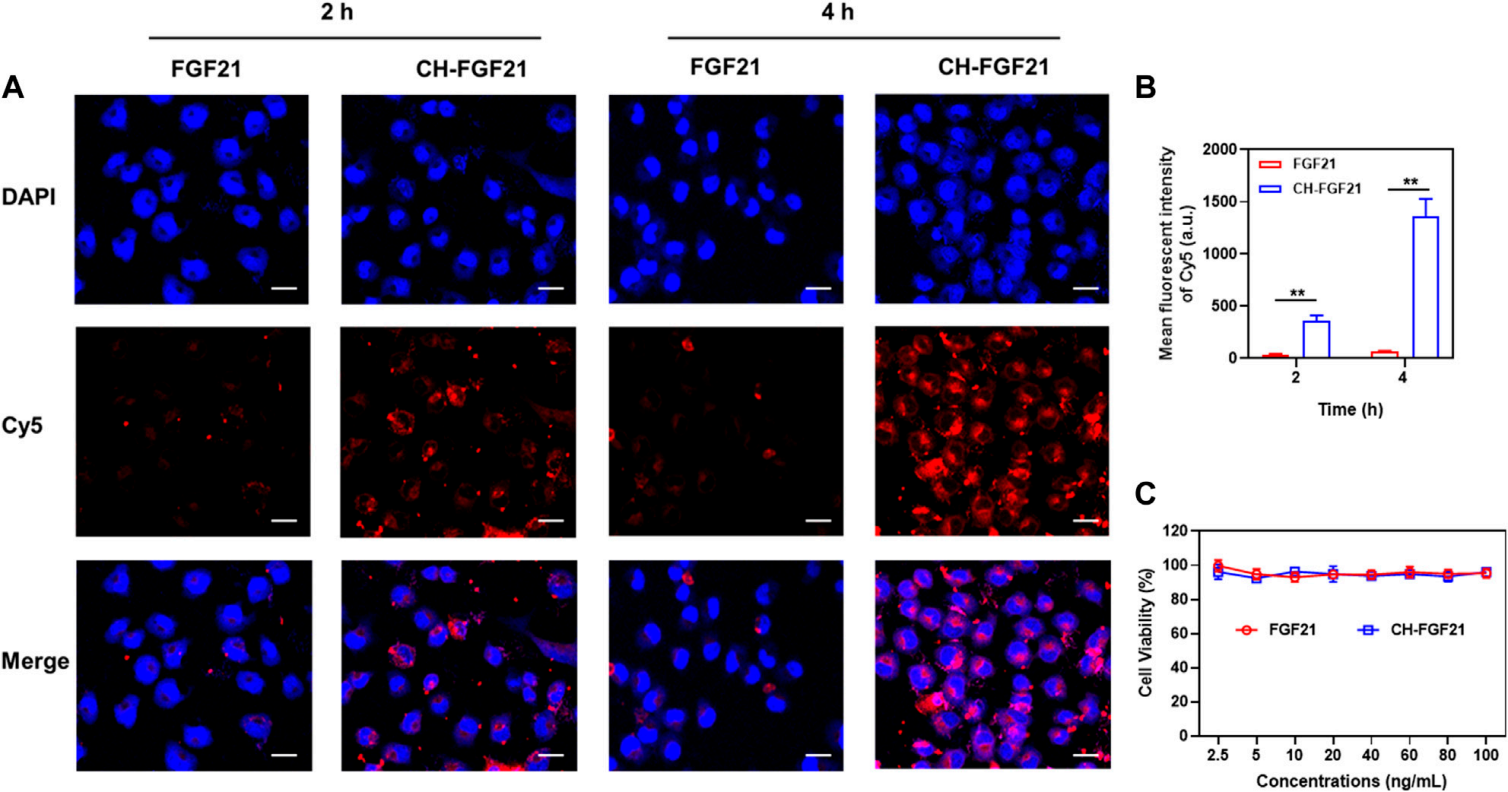
Cellular uptake and cytocompatibility of CH-FGF21. **(A)** Confocal fluorescence images of AML12 cells incubated with FGF21 (100 ng/mL) or CH-FGF21 (100 ng/mL) after 2 h and 4 h. Scale bar = 20 μm. **(B)** Mean fluorescence intensity of each group. Viability of AML12 cells after incubation with different concentrations of **(C)** FGF21 or CH-FGF21 for 24 h. Data are the mean ± SD (*n* = 3). *p ≤ .05, **p ≤ .01.

To investigate the cellular delivery of CH-FGF21, AML12 cells were incubated with Cy5-labeled FGF21 or -CH-FGF21 for 2 h or 4 h, respectively. After treatment, intracellular fluorescence was observed by confocal laser scanning microscopy. The FGF21 group showed extremely low intracellular fluorescence intensity (Figures 2A, B). In contrast, the CH-FGF21 group exhibited considerably higher fluorescence intensity. These data indicated that FGF21 alone had difficulty entering cells during culture, whereas CH-FGF21 enhanced FGF21 uptake into cells effectively. With an increase in incubation duration, the fluorescence intensity of the FGF21 group did not increase significantly, which implied that cells could not uptake FGF21 efficiently; in the CH-FGF21 group, the intracellular fluorescence signal was much higher at 4 h of incubation than that at 2 h of incubation. Hence, AML12 cells could internalize CH-FGF21 efficiently *in vitro*. The CCK-8 assay was carried out to detect the cytotoxicity of CH-FGF21. AML12 cells were treated with FGF21 (2.5–100 ng/mL) or CH-FGF21 (2.5–100 ng/mL), respectively, for 24 h. The cell viability of all treatment groups was >90% with increasing concentrations of FGF21 or CH-FGF21 (Figure 2C), indicating that FGF21 and CH-FGF21 were not toxic to AML12 cells.

### 3.3 Cytoprotective capacity of CH-FGF21

**FIGURE 3:**
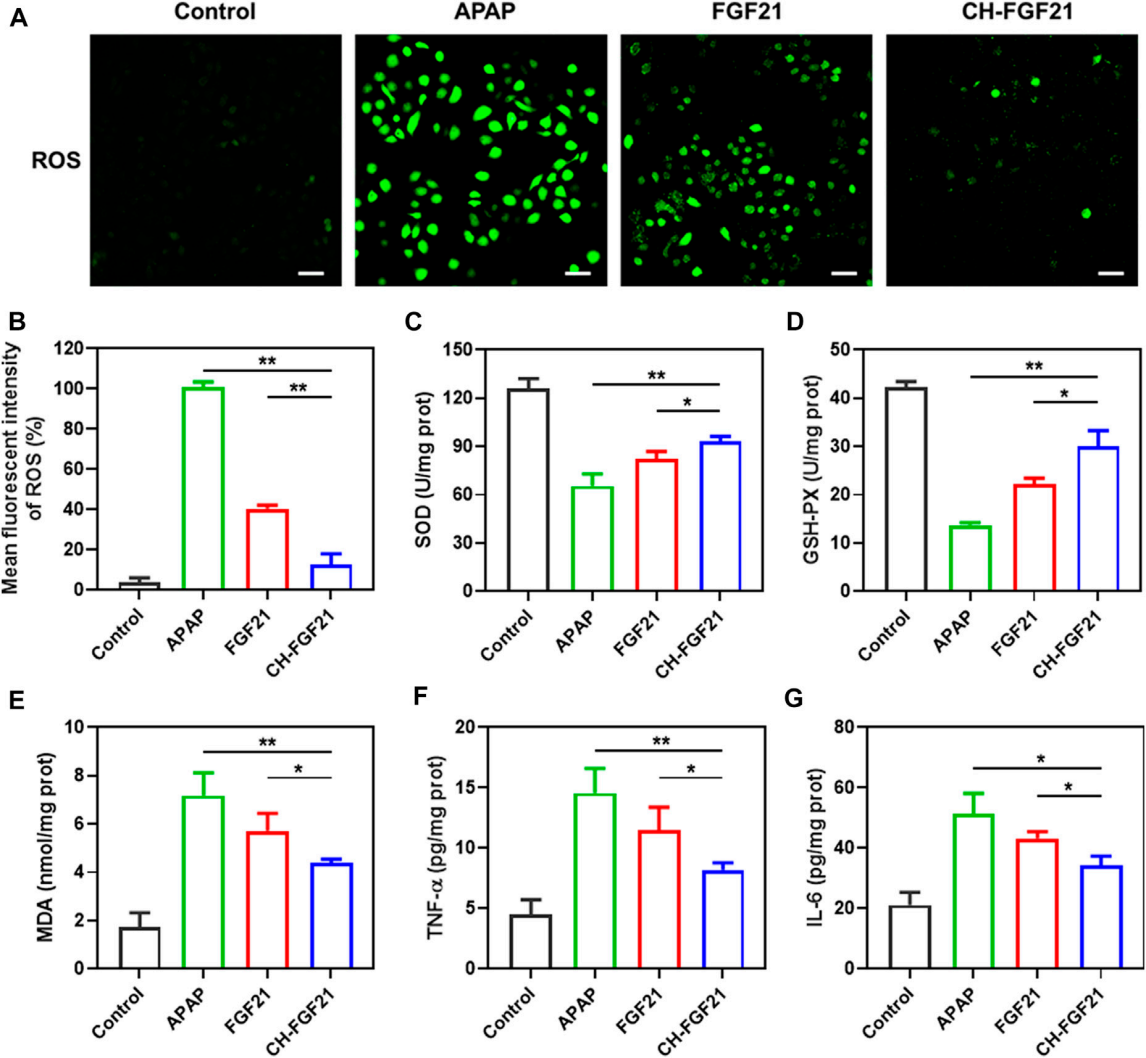
Hepatoprotective properties of CH-FGF21. **(A)** Intracellular reactive oxygen species (ROS) of acetaminophen (APAP)-stimulated AML12 cells after treatment with FGF21 (100 ng/ml) or CH-FGF21 (100 ng/ml) for 24 h. Scale bar = 100 μm. **(B)** Mean fluorescence intensity of ROS. Levels of **(C)** SOD, **(D)** GSH-PX, **(E)** MDA, **(F)** TNF-α, and **(G)** IL-6 in AML12 cells of each group. Data are the mean ± SD (*n* = 3). *p ≤ .05, **p ≤ .01.

An excessive amount of APAP induces a large amount of NAPQI, which exhausts the GSH level in a cell. This phenomenon leads to an imbalance in the intracellular redox state and ROS generation (Zhou et al., 2021). APAP was used to stimulate AML12 cells to ascertain the antioxidant potential of CH-FGF21 on hepatocytes. A fluorescent probe was used to measure intracellular ROS production. Compared with the control group, the ROS level was increased abnormally in the APAP group (Figures 3A, B), implying that APAP caused severe oxidative damage to hepatocytes. In the APAP group, the activity of the intracellular antioxidant enzymes SOD and GSH was reduced whereas MDA generation increased (Figures 3C–E), thereby indicating that the antioxidant defense system of APAP-injured cells was disrupted considerably. The intracellular GSH level in hepatocytes was measured at different time points after APAP stimulation. At different time points, the control group did not show a significant change in the GSH level, whereas the APAP group showed a gradual decrease in the GSH level with sustained APAP damage, and the decreasing trend of intracellular GSH level was decelerated under intervention by CH-FGF21 (Supplementary Figure S2). After stimulation with APAP, damaged AML12 cells produced excessive amounts of ROS, which activated the nuclear factor-kappa B (NF-κB) signaling pathway and domain (NOD)-like receptor protein 3 (NLRP3) inflammasome, causing a massive production of the proinflammatory cytokines TNF-α and IL-6 (Figures 3F, G). Interventions with FGF21 and CH-FGF21 decreased APAP-induced inflammation in AML12 cells and macrophages, thereby suppressing proinflammatory factor (TNF-α and IL-6) release (Supplementary Figure S3). These interventions decreased intracellular generation of ROS effectively and restored the activity of the antioxidant enzyme while reducing the production of the harmful substances MDA, TNF-α, and IL-6 in AML12 cells. These findings demonstrated that CH-FGF21 had superior cytoprotective effects than free FGF21, as well as higher antioxidant and anti-inflammatory capabilities.

### 3.4 *In vivo* distribution of CH-FGF21

**FIGURE 4:**
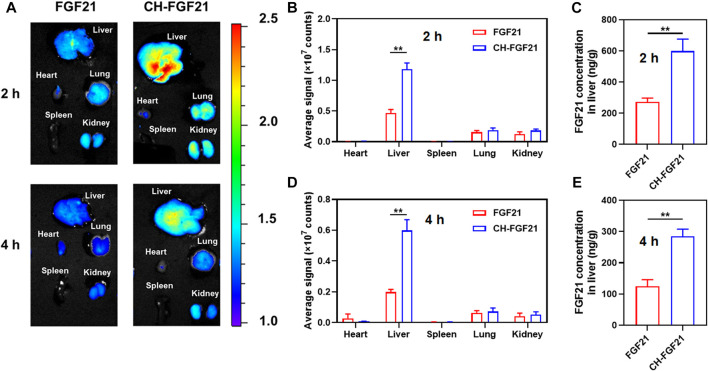
Biodistribution of CH-FGF21. **(A)** Fluorescence images of various organs in mice suffering from acute liver injury (ALI) after intravenous administration of FGF21 or CH-FGF21. Quantitative analysis of fluorescence intensity in the major organs of mice **(B)** 2 h and **(D)** 4 h after injection. FGF21 level in the liver of mice **(C)** 2 h and **(E)** 4 h after injection. Data are the mean ± SD (*n* = 8). *p ≤ .05, **p ≤ .01.

After intravenous injections of fluorescent-labeled FGF21 or CH-FGF21 into ALI mice, the distribution of FGF21 and CH-FGF21 was observed using an imaging system. Fluorescence signals in the liver of mice in the CH-FGF21 group were more intense than those in the FGF21 group 2 h and 4 h after injection (Figure 4A). Quantitative analysis of fluorescence intensity also revealed that CH-FGF21 had good targeting of the liver (Figures 4B, D). More FGF21 accumulated in the liver of mice from the CH-FGF21 group than that of mice in the FGF21 group (Figures 4C, E). This high hepatic accumulation of CH-FGF21 was favorable for ALI therapy.

### 3.5 Therapeutic efficacy of CH-FGF21 in the ALI model

**FIGURE 5:**
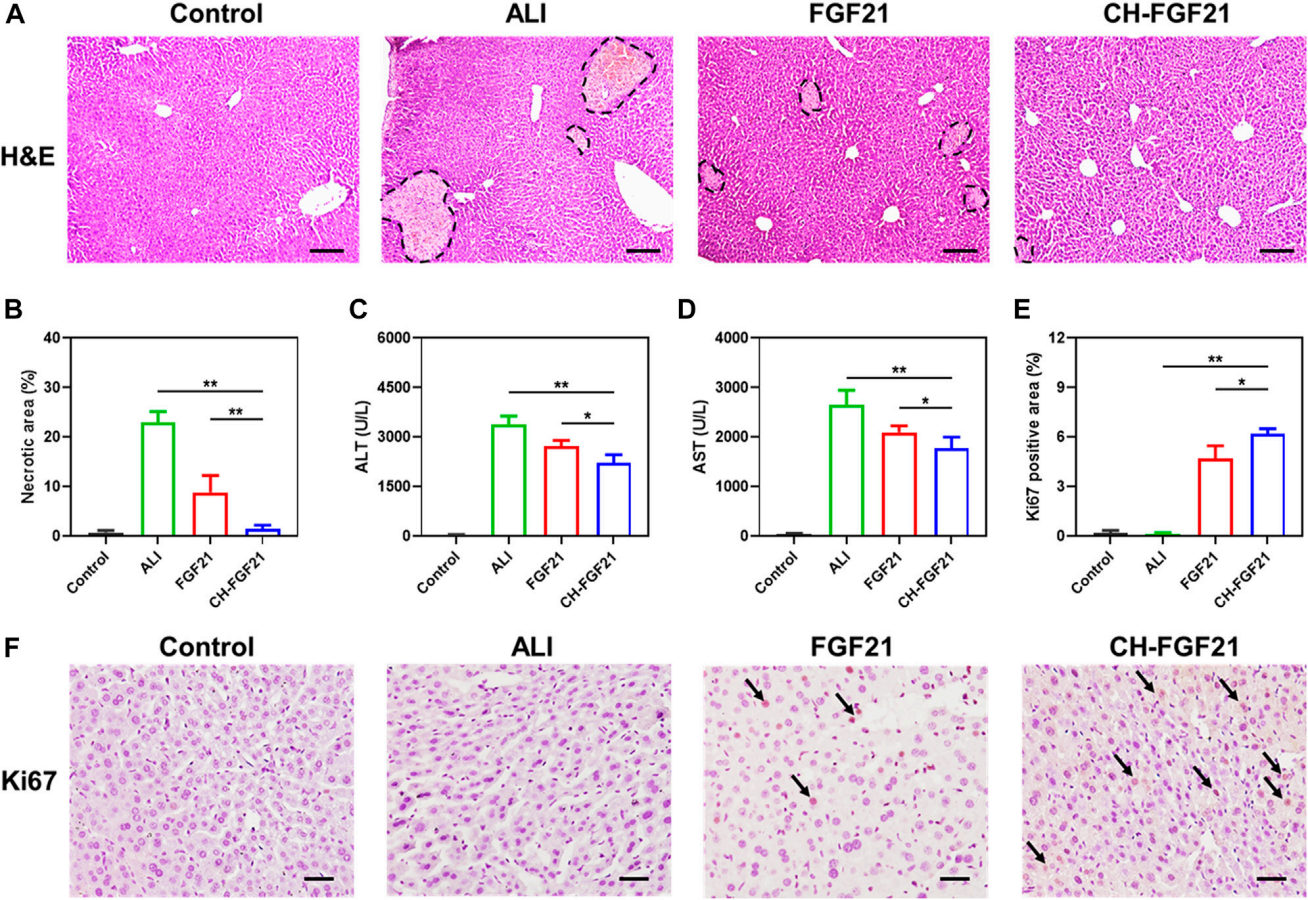
Protective effects of CH-FGF21 on ALI mice. **(A)** Hematoxylin and eosin (H&E) staining of liver tissues. Scale bar = 100 μm. **(B)** Necrotic area in liver tissue. Levels of **(C)** alanine aminotransferase (ALT) and **(D)** aspartate aminotransferase (AST) in the blood of mice in each group 24 h after treatment. **(E)** Quantification of the positive area of Ki67 according to immunohistochemistry. **(F)** Immunohistochemical staining of Ki67 in liver tissue. Scale bar = 50 μm. Data are the mean ± SD (*n* = 8). *p ≤ .05, **p ≤ .01.

Based on the good antioxidative activity and preferable biodistribution of CH-FGF21, its therapeutic potential against APAP-induced ALI in mice was investigated. H&E staining of liver tissues was undertaken to evaluate pathological changes in various groups. Hepatocyte nuclei were intact, and the morphology of liver lobules was visible in the normal group. A substantially damaged region of liver tissue and necrotic areas were found in the ALI group, along with severe tissue disruption of architecture, nucleus deformation, and an aberrant vacuole-like structure (Figure 5A). After FGF21 therapy, liver injury improved significantly, but necrosis and other pathological changes were visible. Little obvious damage was observed in the liver tissues of mice in the CH-FGF21 group; they had histological features similar to the liver tissues of mice in the control group, thereby reflecting the superior hepatoprotective effects of CH-FGF21 compared with those of FGF21 (Figure 5B). The common clinical indicators of liver function (ALT, AST) in the blood of mice of each group were measured (Figures 5C, D). Due to the hepatotoxic effects of APAP, hepatocytes were severely damaged, which caused a massive release of intracellular enzymes into the blood, resulting in a dramatic increase in the levels of ALT and AST in ALI mice. In terms of AST and ALT levels, NAC showed the best effects for the maintenance of liver function in all treatment groups, whereas intervention with CH nanoparticles was virtually ineffective (Supplementary Figure S5). Under the intervention of FGF21 or CH-FGF21, the levels of ALT and AST declined. In comparison with FGF21 therapy, CH-FGF21 administration inhibited the levels of AST and ALT released from hepatocytes into blood significantly during ALI progression (Supplementary Figure S4). Hence, CH-FGF21 was more effective in protecting hepatocytes and liver tissue from APAP-related damage. Ki67 expression was barely observed in the liver of mice in the control group and ALI group. Ki67 expression was present in the liver of mice in the FGF21 group and particularly in the CH-FGF21 group, thereby indicating that CH-FGF21 could regulate regeneration of injured liver tissue (Figures 5E, F).

### 3.6 Anti-inflammatory and antioxidant effects of CH-FGF21 in an ALI model

**FIGURE 6:**
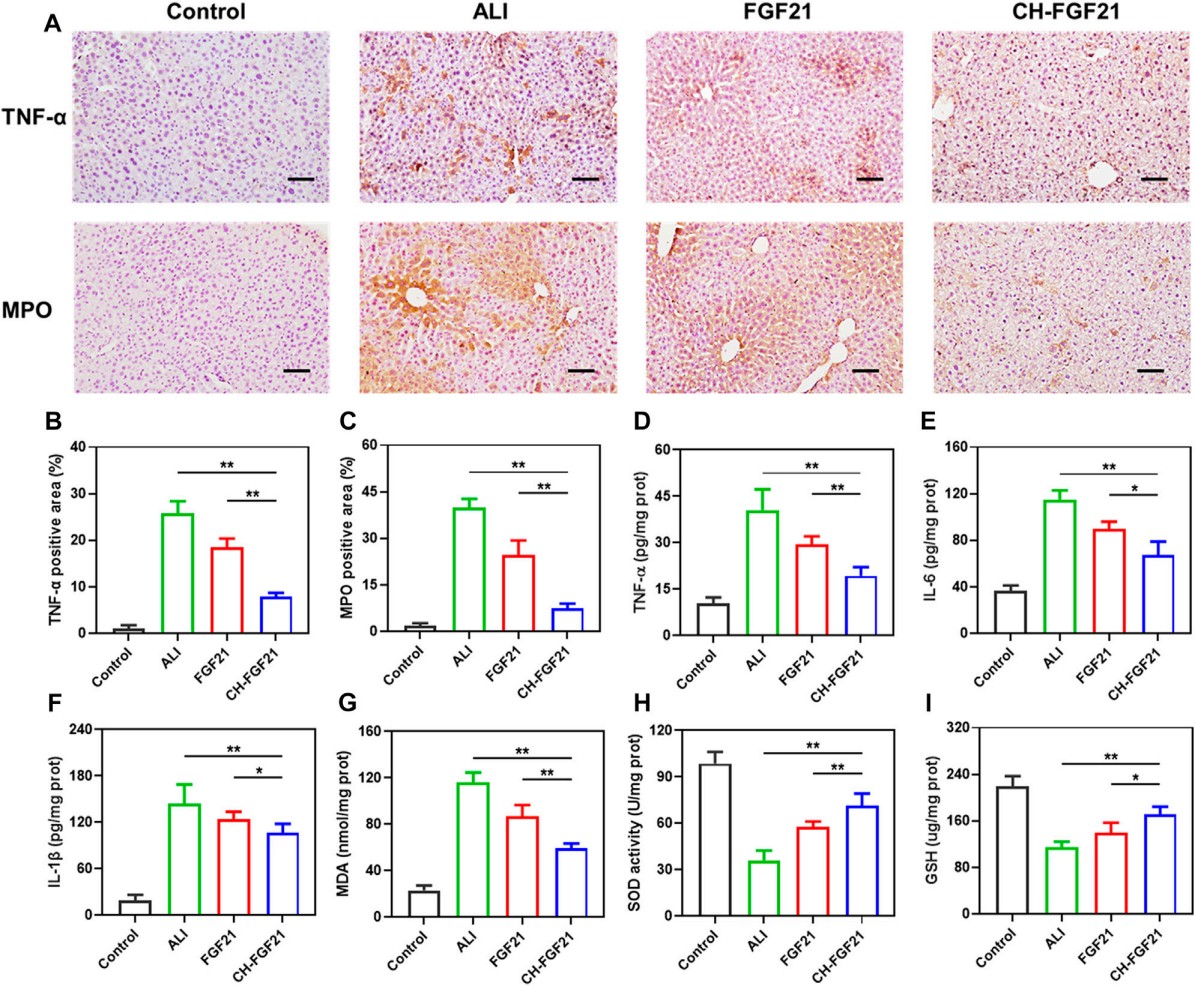
Evaluation of inflammation and oxidative stress in liver tissues. **(A)** Immunohistochemical staining of tumor necrosis factor (TNF)-α and myeloperoxidase (MPO) in liver tissue. Scale bar = 100 μm. Quantification of the positive area of **(B)** TNF-α and **(C)** MPO after immunohistochemical staining of liver tissue. Levels of **(D)** TNF-α, **(E)** IL-6, **(F)** IL-1β, **(G)** MDA, **(H)** SOD, and **(I)** GSH in the liver tissue of mice in each group. Data are the mean ± SD (*n* = 8). *p ≤ .05, **p ≤ .01.

Given the importance of inflammation and oxidative stress in damage to liver tissue, we investigated the anti-inflammatory and antioxidant efficacy of CH-FGF21 in an ALI model. Immunohistochemical analyses showed that the liver of ALI mice had high expression of TNF-α and MPO, production of the harmful proinflammatory molecules TNF-α, IL-6, IL-1β, and MDA was increased markedly, and the levels of SOD and GSH were decreased. Hence, APAP induced severe inflammatory and oxidative damage to the liver. The liver of ALI mice improved significantly after therapy with FGF21 or CH-FGF21, with the latter demonstrating superior anti-inflammatory and antioxidant activity. Immunohistochemical analyses revealed a considerable reduction in expression of TNF-α and MPO (Figures 6A–C), CH-FGF21 therapy suppressed the secretion of proinflammatory factors (Figures 6D–F), reduced the production of MDA (Figure 6G) and MPO (Supplementary Figure S6), and increased the levels of antioxidants in the liver (Figures 6H, I). These data suggested that CH-FGF21 could help to alleviate oxidative stress and inflammatory reactions in ALI mice. Systemic administration of CH-FGF21 led to preferential localization in the liver. Hence, CH-FGF21 with liver-targeted function transported more FGF21 to the injured liver, and higher concentrations of FGF21 accumulated in the liver of ALI mice injected with CH-FGF21, which demonstrated the superiority of CH-FGF21 in the treatment of ALI.

## 4 Discussion

NAPQI is produced by the metabolism of APAP. NAPQI can cause abnormal changes in the mitochondrial morphology and function of hepatocytes (Ramachandran and Jaeschke, 2020). NAPQI can also interfere with the transcription and expression of genes associated with antioxidants in hepatocytes, disrupt hepatic homeostasis, and aggravate liver injury. Sustained injury to and necrosis of hepatocytes lead to the activation and aggregation of large amounts of proinflammatory mediators (Chowdhury et al., 2020). Upon activation of inflammation-related signaling pathways, proinflammatory cytokines cause hepatocyte apoptosis and inflammatory responses, which lead to abnormal liver function (Bai et al., 2021; He et al., 2022).

FGF21 is an endocrine hormone involved in the metabolism of glucose and lipids as well as energy balance. APAP overdose-induced liver injury has been shown to be exacerbated significantly in FGF21-knockout mice, and that FGF21 promotes expression of peroxisome proliferator-activated receptors in the liver. These actions lead to upregulation of expression of the antioxidant stress transcription factor Nrf2 and other antioxidant genes, which inhibits activation of the NF-κB signaling pathway, thereby reducing oxidative stress-related hepatic injury (Ye et al., 2014). FGF21 has considerable promise in the therapy of liver illnesses, but its medicinal applicability is hampered severely by its poor bioavailability, short half-life, rapid degradation, and non-specific organ targeting (Gao et al., 2021).

The major methods employed to extend the half-life of FGF21 are modification of polyethylene glycol, polypeptide fusion, and fusion of the Fc domain of an antibody (Ye et al., 2013; Yin et al., 2016). However, these approaches have not addressed the suboptimal *in vivo* distribution of FGF21. Furthermore, several studies have focused on FGF21 fusion with other targets, such as dual-target agonists with FGF21 and glucagon-like peptide-1 (Pan et al., 2021). Chemically modified or ligand-functionalized DDSs with active targeting have also been developed for the treatment of liver diseases (Bottger et al., 2020). Despite their potent targeting abilities, the safety, tolerability, pharmacokinetics, and pharmacodynamics of such DDSs have not been explored extensively.

Chitosan is a renewable natural cationic polysaccharide. It is used widely in food, pharmaceutical, and biomedical applications due to its potent biological properties. As a drug-delivery vehicle, chitosan can be synthesized and characterized readily, as well as being biocompatible, biodegradable, bioadhesive, non-immunogenic, non-toxic, and water-soluble. Chitosan nanoparticle-based DDSs can improve the stability, control the release, and enhance the cellular uptake of a drug (Huang et al., 2017; Mikusova and Mikus, 2021). Drug transport into a cell is a key step for drugs to exert their effects. Chitosan can interact with proteoglycans on the cell surface in non-specific and electrostatic manners, as well as between ligands of chitosan and receptors on the cell membrane surface. These actions facilitate the transmembrane transport and intracellular entry of drugs (Aibani et al., 2021). Our results on cellular uptake showed that hepatocytes internalized CH-FGF21 more readily than free FGF21, which might be attributed to the properties of chitosan. The efficient uptake of FGF21 by cells facilitated the pharmacological effects of FGF21, and the hepatocytes in the CH-FGF21 group showed strong resistance to APAP damage.

As a highly biocompatible and effective delivery system, CH nanoparticles have been prepared for use for the delivery of bovine serum albumin, oligonucleotides, and nerve growth factor (Liu et al., 2007; Li et al., 2017; Pilipenko et al., 2019). We revealed that CH nanoparticles could also transport FGF21 efficiently *in vivo*. By electrostatic interaction, the positively charged CH nanoparticles could bind readily to negatively charged FGF21 and then self-assemble to form CH-FGF21 with a nanosphere structure. Due to passive targeting and EPR effects, CH-FGF21 could accumulate specifically in APAP-injured liver tissue, thereby enhancing the local therapeutic concentration of FGF21. In addition, CH-FGF21 circulated longer than free FGF21 in blood, and the blood circulation delivered CH-FGF21 to the inflamed liver. As a result, CH-FGF21 reduced pathological changes in the liver, including the necrosis and apoptosis of hepatocytes in central hepatic lobules. Meanwhile, the levels of liver function-related indices in mice of the CH-FGF21 group were reduced, thereby indicating that CH-FGF21 therapy restored normal hepatic function in our ALI model. Compared with FGF21 therapy, CH-FGF21 treatment suppressed the production of proinflammatory cytokines and increased the activity of antioxidant enzymes more effectively, which halted ALI development. Overall, these *in vivo* results suggested that CH-FGF21 amplified the therapeutic effects of FGF21 against ALI.

CH-FGF21 had good biocompatibility, but the passive-targeting effect of these nanoparticles alone was not sufficient. Therefore, CH-FGF21 could be optimized and modified to enhance the liver-targeting effect in subsequent research. The therapeutic effects and mechanism of action of CH-FGF21 in the treatment of chronic hepatopathy (e.g., fatty liver) could also be investigated.

## 5 Conclusion

We developed biocompatible, liver-targeting nanoparticles: CH-FGF21. We illustrated the potential of CH-FGF21 as anti-inflammatory and antioxidant agents for ALI therapy. *In vitro* results showed that CH-FGF21 enhanced the uptake efficiency of FGF21 by hepatocytes and reduced APAP-induced damage to hepatocytes. Due to the intrinsic passive targeting of the liver and EPR effect of nanoparticles, CH-FGF21 accumulated preferentially in the inflamed liver of ALI mice. With this advantage, CH-FGF21 increased the hepatic tolerance to inflammatory and oxidative damage while decreasing the detrimental effects of APAP on liver tissue.

## Data Availability

The original contributions presented in the study are included in the article/Supplementary Material, further inquiries can be directed to the corresponding authors.
