# Supplementary material for: Self-assembled FGF21 nanoparticles alleviate drug-induced acute liver injury
Source: Front Pharmacol. 2023 Jan 10;13:1084799. doi: 10.3389/fphar.2022.1084799 (PMC9871310; doi:10.3389/fphar.2022.1084799)
Supplement: Supplementary file 1 [file DataSheet1.docx]

Supplementary Material

Self-Assembled FGF21 Nanoparticles Alleviate Drug-Induced Acute Liver Injury

**Figure S1.** In vitro release of CH-FGF21 in 120 h. Data are expressed as the mean ± SD (n = 3).

**Figure S2.** The level of GSH in hepatocytes of each group after different time points of APAP stimulation. Data are expressed as the mean ± SD (n = 3).


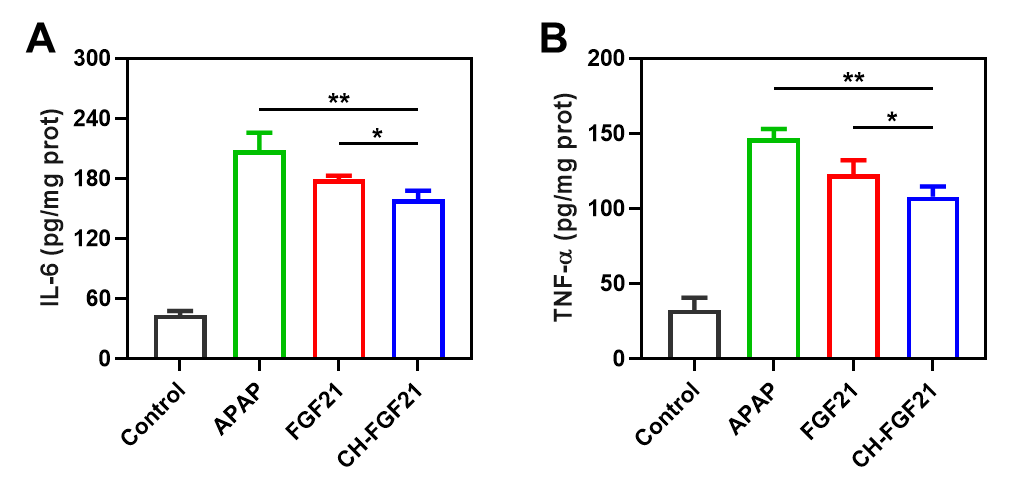


**Figure S3.** The levels of (A) IL-6 and (B) TNF-α in the macrophages of each group. Data are expressed as the mean ± SD (n = 3). *P ≤ 0.05, **P ≤ 0.01.


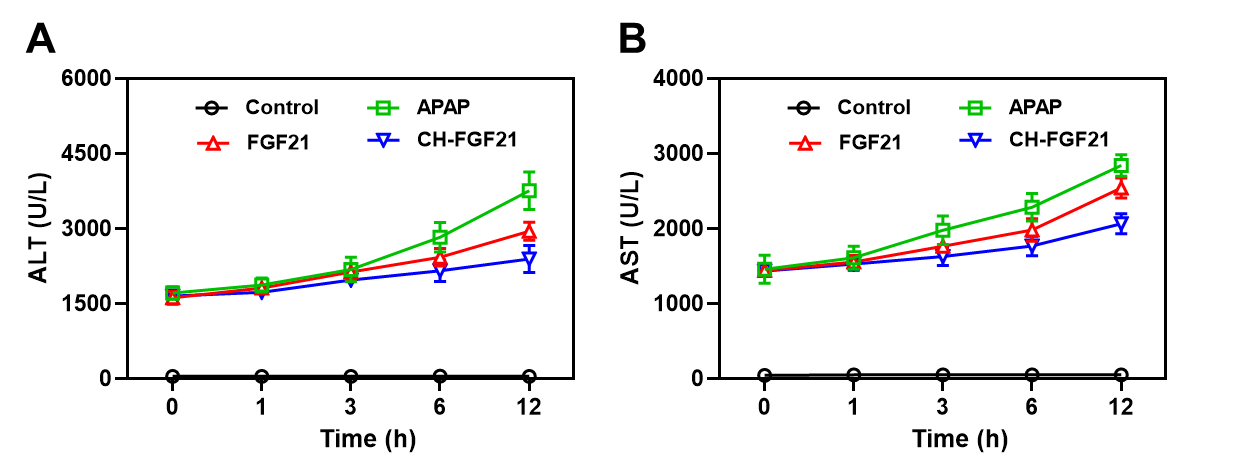


**Figure S4.** The levels of (A)ALT and (B) AST of each group after treatment at different time points. Data are expressed as the mean ± SD (n = 8).


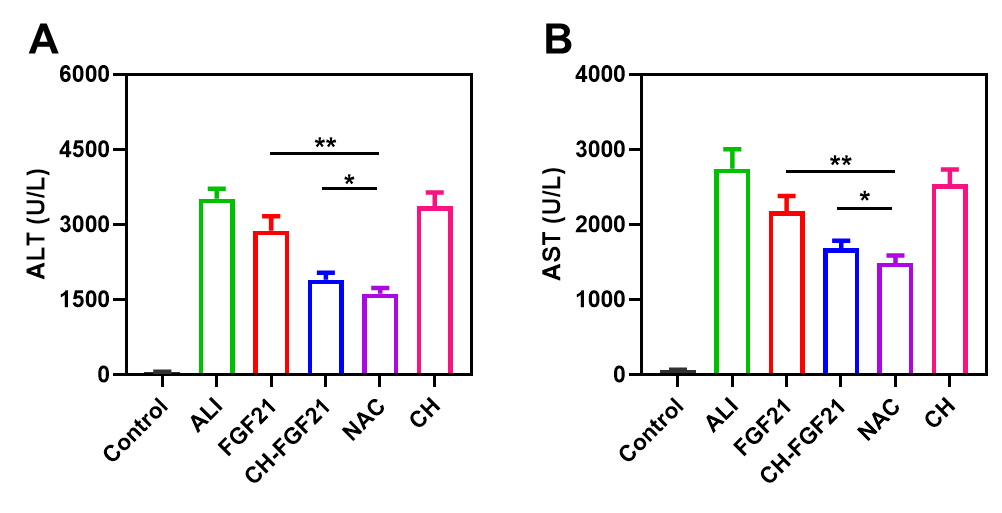


**Figure S5.** The levels of (A) ALT and (B) AST of each group. Data are expressed as the mean ± SD (n = 8). *P ≤ 0.05, **P ≤ 0.01.


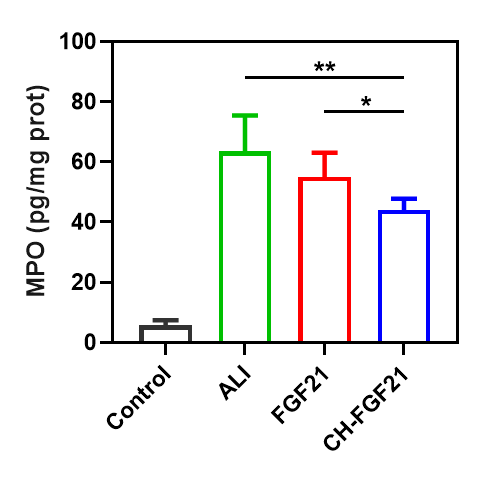


**Figure S6.** The levels of MPO in the liver tissue of each group. Data are expressed as the mean ± SD (n = 8). *P ≤ 0.05, **P ≤ 0.01.
